# Toxicity Mitigation of Textile Dye Reactive Blue 4 by Hairy Roots of *Helianthus annuus* and Testing Its Effect in *In Vivo* Model Systems

**DOI:** 10.1155/2022/1958939

**Published:** 2022-07-25

**Authors:** Kanchanlata Tungare, Rinkey Shahu, Vyankatesh Zambare, Payal Agarwal, Renitta Jobby, Nazima Nisar, Nadiyah M. Alabdallah, Fatimah A. Al-Saeed, Parul Johri, Sachidanand Singh, Mohd Saeed, Pamela Jha

**Affiliations:** ^1^School of Biotechnology and Bioinformatics, D. Y. Patil Deemed to be University, Plot No. 50, Sector 15, CBD Belapur, 400614 Navi Mumbai, Maharashtra, India; ^2^Amity Institute of Biotechnology, Amity University Maharashtra, Mumbai Pune Expressway, Bhatan, Post Somatane, Panvel, Mumbai, Maharashtra 410206, India; ^3^Huck Institutes of the Life Sciences, The Pennsylvania State University, University Park PA 16802, USA; ^4^Amity Centre of Excellence in Astrobiology, Amity University, Maharashtra-Pune Expressway, Bhatan, Panvel, Mumbai, Maharashtra 410206, India; ^5^Department of Clinical Laboratory Sciences, College of Applied Medical Sciences, King Khalid University, Abha, Saudi Arabia; ^6^Department of Biology, College of Science, Imam Abdulrahman Bin Faisal University, P.O. Box 1982, 31441 Dammam, Saudi Arabia; ^7^Department of Biology, College of Science, King Khalid University, Abha, Saudi Arabia; ^8^Department of Biotechnology, Dr. Ambedkar Institute of Technology for Handicapped, Kanpur 208001, India; ^9^Department of Biotechnology, Smt. S. S. Patel Nootan Science & Commerce College, Sankalchand Patel University, Visnagar, 384315 Gujarat, India; ^10^Department of Biology, College of Sciences, University of Hail, Hail, Saudi Arabia; ^11^Department of Biological Sciences, Sunandan Divatia School of Science, NMIMS Deemed to be University, Vile Parle (West), Mumbai 400056, India

## Abstract

An anthraquinone textile dye, Reactive Blue 4 (RB4), poses environmental health hazards. In this study, remediation of RB4 (30-110 ppm) was carried out by hairy roots (HRs). UV-visible spectroscopy and FTIR analysis showed that the dye undergoes decolourization followed by degradation. In addition, toxicity and safety analyses of the bioremediated dye were performed on *Allium cepa* and zebrafish embryos, which revealed lesser toxicity of the bioremediated dye as compared to untreated dye. For *Allium cepa*, the highest concentration, i.e., 110 ppm of the treated dye, showed less chromosomal aberrations with a mitotic index of 8.5 ± 0.5, closer to control. Two-fold decrease in mortality of zebrafish embryos was observed at the highest treated dye concentration indicating toxicity mitigation. A higher level of lipid peroxidation (LPO) was recorded in the zebrafish embryo when exposed to untreated dye, suggesting a possible role of oxidative stress-inducing mortality of embryos. Further, the level of LPO was significantly normalized along with the other antioxidant enzymes in embryos after dye bioremediation. At lower concentrations, mitigated samples displayed similar antioxidant activity comparable to control underlining the fact that the dye at lesser concentration can be more easily degraded than the dye at higher concentration.

## 1. Introduction

The textile industry is one of the major industries using dyes of various kinds. Nearly 80,000 tonnes of dyestuff are produced in India, and approximately 10,000 textile dyes are manufactured commercially on a global scale. Globally, the production of textile dye is 7 × 10^5^ metric tons, of which 30% of dyes are used superfluously [1]. 2% of these dyes fail to adhere to fibre and consequently discharge into effluents, implying that the textile dyeing process generates harmful wastewater [2]. The most frequently used dyeing compounds are azo dyes followed by anthraquinone dyes owing to their stability against microbial degradation and photolysis [3, 4]. Azo groups and aromatic groups of anthraquinone dyes are resistant to chemical degradation and are liable to accumulate in the environment with a very high degree of persistence [1]. Anthraquinone dyes are the second most abundantly produced dyes on a global scale. While numerous groups have investigated the remediation of azo dyes, less attention has been dedicated to the degradation of anthraquinone dyes [5]. Industrial effluents containing these substances have a detrimental influence on natural water bodies and aquatic life, while also offering an implicit hazardous and even carcinogenic risk to humans [6]. Therefore, amelioration of these effluents is imperative and research on it is a pressing priority.

Existing methods for textile wastewater treatment include physical and chemical, in addition to certain engineered techniques such as adsorption, electrolysis, oxidation, and photoionization. The majority of the procedures discussed above have significant disadvantages, including high costs, low efficiency, and the generation of hazardous intermediates [7]. Adsorption, on the other hand, requires less land space, offers more flexibility in the design and operation, generates fewer toxic elements, and extends increased removal of contaminants. However, it leads to concentration of pollutants from textile effluents to the adsorbent and does not ensure complete removal of the pollutant. Therefore, it is of importance to determine if the plant species are capable of transforming or degrading the dye into simpler, nontoxic products. Hairy roots (HRs) from various plant species are extensively used to assess tolerance, accumulation, and/or elimination of environmental pollutants [8, 9]. Additionally, HR crops also serve as *in vivo* model systems to investigate phytoremediation processes and mechanisms [10]. Amidst all the pollutants that endanger biodiversity, industrial dye-based effluents present the most serious threat [11]. Textile colours in extremely low quantities in effluent and their byproducts are likewise hazardous to the ecology [12]. Hence, only the adsorption process is not enough, and degradation of the dyes also becomes critical. Numerous researchers have described the use of HRs to degrade dyes; however, there is limited data investigating the safe nature of the bioremediated dyes using in vitro models. Among the plants used for remediation of pollutants, sunflower (*Helianthus annuus*) is well known for remediation of pollutants like phenol and heavy metals [13]. Hence, the present study is aimed at evaluating the bioremediation potential of HRs towards an anthraquinone textile dye RB4, and toxicity analysis of the degraded dye products was performed using *in vivo* model systems. The outcomes from this study are expected to further standardize the use of HRs as an effective bioremediation agent for a broad range of textile dyes.

## 2. Materials and Methods

### 2.1. Raw Materials and Chemicals

Hairy roots were obtained from explants of *Helianthus annuus*. The textile dyes RB4, Direct Black B (DBB), Reactive Green 19 (RG19), Reactive Orange 84 (RO84), Reactive Yellow 17 (RY17), Reactive Red 35 (RR35), Reactive Red M8B (RRM8B), Reactive Red M5B (RRM5B), Reactive Violet 5R (RV5R), and Reactive Violet 13 (RV13) were obtained from Appex Industries, Ahmedabad, India. All other chemicals used were of high analytical grade.

### 2.2. Preparation of Hairy Roots

*Agrobacterium rhizogenes* MTCC532 was used for HR induction in leaf explants of *Helianthus annuus*, and molecular confirmation was done by PCR [14].

### 2.3. Decolourization of Textile Dyes

The capacity of HRs to decolourize 10 textile dyes for 120 h was performed, and percent decolourization was calculated by the method of Jha et al. [14] using the following formula:

(1) $$\begin{array}{c} \%\text{Decolourization}=\frac{\text{Initial absorbance}-\text{final absorbance}}{\text{Initial absorbance}}\times 100. \end{array}$$

### 2.4. Effect of Different Parameters on Decolourization of Dyes

The study was carried out by incubating HRs and selected dye (RB4) solution of varying pH (1.8, 2.8, 3.8, 4.8, 5.8, 6.8, and 7.8), temperatures (27 ± 2°C, 35 ± 2°C, and 45 ± 2°C), initial dye concentrations (30, 50, 70, 90, and 110 ppm), and biomass dosages (10-50 g/L). The decolourization (%) was calculated (see Section 2.3) for each sample with varying parameters.

### 2.5. Phytodegradation Analysis of Dyes by HRs

#### 2.5.1. UV-Visible Spectroscopy

The absorbance of the supernatant obtained at 0 h and during decolourization of RB4 by HRs was taken in a UV-Vis spectrophotometer (Shimadzu UV-Vis Spectrophotometer 2800) at 595 nm wavelength.

#### 2.5.2. Fourier Transform Infrared Spectroscopy (FTIR)

The metabolites extracted, after decolourization of RB4, were mixed with spectroscopically pure KBr in the ratio of 5 : 95. The analysis was performed in the mid-IR region of 400-4000 cm^−1^with 16 scan speeds using the PerkinElmer 783 Spectrophotometer and compared with control [14].

### 2.6. Toxicity Analysis

#### 2.6.1. *Allium cepa* Test

The first set of bulbs was exposed to water (control), the second set to untreated dye (110 ppm), and the third set to treated dye (110 ppm) for 120 h. The cells were checked for different types of chromosomal aberrations [15].

#### 2.6.2. Zebrafish Maintenance and Fish Embryo Toxicity (FET) Test

The embryos of wild-type zebrafish were used and maintained as per Westerfield 2000. Fertilized eggs in the cleavage period until the blastula stage were selected under an inverted microscope (Nikon ECLIPSE TS100) for subsequent experiments. An *in vivo* toxicity test was performed as per the Organization for Economic Co-operation and Development (OECD) test guideline no. 236. For this study, healthy zebrafish embryos were placed in 6-well culture plates (15 embryos per well). 30 mL per well samples of each untreated and treated dye was used in the range of 30, 50, 70, 90, and 110 ppm, respectively. Embryos in sterile distilled water were used as a control. The plates were then kept in the dark at 26 ± 2°C. The embryos exposed from 24 to 96 h (every 24 h) were used for toxicity and biochemical analyses. The embryos exposed to the untreated and treated dye were evaluated for hatching, mortality, tail malformations, heartbeat, coagulation, malformation of somites, development of eyes, pigmentation, and edemas [16, 17]. All the experiments were conducted in triplicate.

### 2.7. Biochemical Assays

The embryos were homogenized in an ice-cold buffer (0.1 M Tris-HCl, 0.1 mM EDTA, and 0.1% Triton X-100 (*v*/*v*), pH 7.8). The homogenates were centrifuged, and the supernatants were used for the measurement of total protein and malondialdehyde (MDA) content as per Rajneesh et al. [18], superoxide dismutase (SOD) activity as per Bewley et al. [19], succinate dehydrogenase (SDH) as per Singh et al. [20], catalase (CAT) assay as per Bhori et al. [21], and peroxidase (POX) assay as per Bhunia et al. [22].

### 2.8. Statistical Analysis

Each analysis was performed using GraphPad Prism 8.4.2 in triplicate, and the results were represented as mean ± SD. The significance of the difference among the groups was assessed using a two-way analysis of variance (ANOVA) test followed by Tukey's post hoc test of the difference between all group means. Symbols used for significance are ^∗^*p* < 0.05, ^∗∗^*p* < 0.01, and ^∗∗∗^*p* < 0.001.

## 3. Results and Discussion

### 3.1. Screening of Textile Dyes

**Figure 1:**
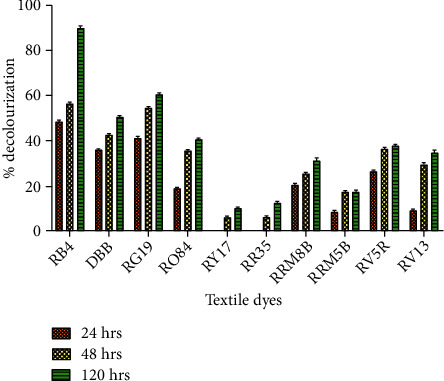
Decolourization of different dyes by hairy roots: untreated dye (U) and treated dye (T).

**Figure 2:**
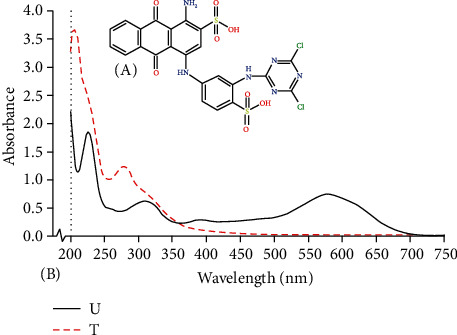
(a) Structure of RB4 and (b) UV-visible spectrum of RB4 untreated dye (U) and treated dye (T).

As the dyes used in the textile processing industry are of varying chemical structures, the effluents from the industry significantly vary to a large extent in composition. Therefore, it was important to evaluate the decolourization efficiency of HRs for different dyes. All the dyes were screened, and decolourization was observed in the range of 10% to 90% (Figure 1) after 24 h, 48 h, and 120 h of incubation. The dye, which showed maximum decolourization, was RB4, i.e., 90% after 120 h (Figure S1). The absorbance of supernatants at 120 h of decolourized RB4 was taken in a UV-visible spectrophotometer and compared with that of an untreated sample (Figure 2(b)). The decolourization percentage varied with different dyes, which might be attributed to their structural differences [23], increased structural complexities due to high molecular weight, and occurrence of inhibitory groups such as NO_2_ and SO_3_Na [24].

### 3.2. Effect of Different Parameters on Dye Decolourization by HRs

Maximum decolourization was observed at pH 4.8-5.8 (Figure S2), while temperature from 25 to 45°C (Figure S3) did not substantially affect decolourization. Therefore, all further experiments were conducted at 25°C and medium pH 5.8. Maximum decolourization (>90%) was observed at a biomass dosage of 40 g/L and 50 g/L (Figure S4). Decolourization was observed to be inversely proportional to the dye concentration ranging 30-110 ppm. The lowest concentration decolourized to 99%, and the highest was 51% (Figure S5).

### 3.3. FTIR

**Figure 3:**
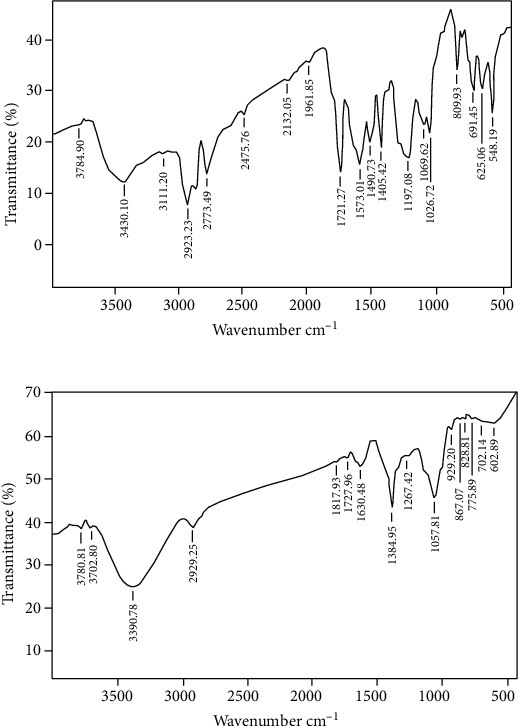
FTIR spectrum of RB4: (a) untreated dye and (b) treated dye.

The FTIR spectrum of untreated and treated samples showed a variation in the molecular structure which is due to biodegradation of dye (Figure 3). In untreated dye, band at 3430.10, 2132.05, 1020-1220, and 691.45-548.19 cm^−1^ represents O–H stretching vibration of hydrogen-bonded hydroxyl groups in polymeric association, C=C stretching bond of alkynes molecule, alkyl amine, and halogen compound (chloro compound) (C–CI), respectively. Similar broad bands at 3420.87 and 3442.87 cm^−1^, respectively, representing –NH– and hydroxyl (–OH) extensions in the RB4 spectrum were also reported by Afreen et al. [25] and Atteke et al. [26]. In our study, FTIR spectrum of treated dye showed bands at 3390.78, 1727.96, 1630.48, and 702.14-602.89 cm^−1^ which are attributed to the presence of bonded N–H/C–H/O–H stretching of amines and amides, ketones, aromatic ring (C=C in plane) stretching symmetric, and halogen compound (C–CI), respectively. In another study, the difference in bands obtained in FTIR spectra of Reactive Red 198 and after its decolourization by HRs of *Tagetes patula* also indicated the degradation of the dye [27]. In a previous study, the FTIR spectra of another azo dye, Reactive Green 19A, also indicated similar functional groups like sulfonic groups and azo groups at the same wavenumbers as shown in our results [11]. Also, our previous study had reported the presence of azo groups in parent dye Acid Red 114 and the absence of these groups along with the emergence of new bands in treated dye by HRs of *Ipomoea carnea*, thereby suggesting the degradation of dye [28].

### 3.4. Toxicity Analysis

#### 3.4.1. *Allium cepa* Test

**Figure 4:**
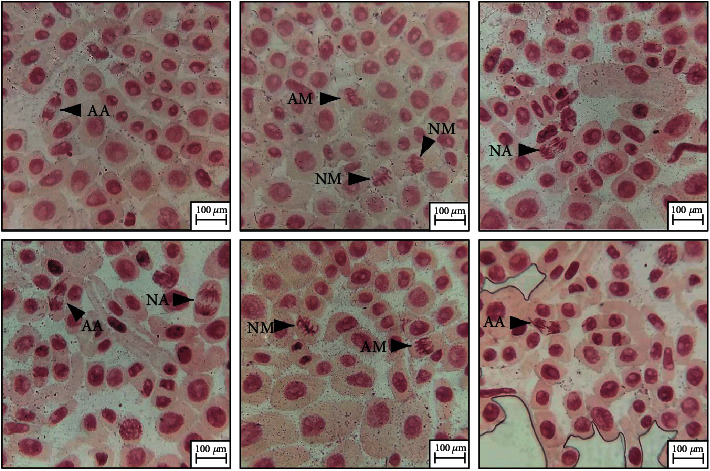
Stages of mitosis in root tips of *Allium cepa*: NM: normal metaphase; AM: abnormal metaphase; NA: normal anaphase; AA: abnormal anaphase.

Higher plants are recognized as excellent genetic models to detect environmental mutagens and are frequently used in monitoring studies. Among the plant species, *Allium cepa* has been used to evaluate DNA damage, such as chromosome aberrations and disturbances in the mitotic cycle. Meristematic mitotic cells of *A. cepa* are established as capable constituents for cytotoxicity analysis [29]. In the present study, cytotoxic implications of treated and untreated dye were analyzed based on the mitotic index (MI) and chromosomal aberrations (Table 1). The MI of bulbs grown in 110 ppm untreated dye was found to be 6.5 ± 0.15, which is statistically lower than cells in distilled water, whereas the MI of bulbs grown in 110 ppm treated dye was found to be improved and significantly closer to distilled water samples. Likewise, the percentage of aberrant cells at 110 ppm untreated dye was statistically higher (*p* < 0.001) than that of distilled water cells and significantly decreased to 10% in the case of cells exposed to the treated dye. In another study, toxicity analysis of the treated textile dye, RR35, using *A. cepa* root cells demonstrated improvement in cell viability, root length, mitotic index, and chromosomal aberrations when compared to untreated dye [30]. Different types of chromosomal abnormalities in treated and untreated dye samples like sticky metaphase, disturbed metaphase, anaphasic bridge, disturbed anaphase, and laggards have been previously reported [31]. Our microscopic results showed a significantly higher number of cell alterations in untreated than in control, with the most common being binucleate cells (Figure 4). In addition, untreated dye cells are shown to have more laggards than the cells exposed to treated dye, which is indicative of the genotoxic nature of the nonremediated dye.

#### 3.4.2. FET

**Figure 5:**
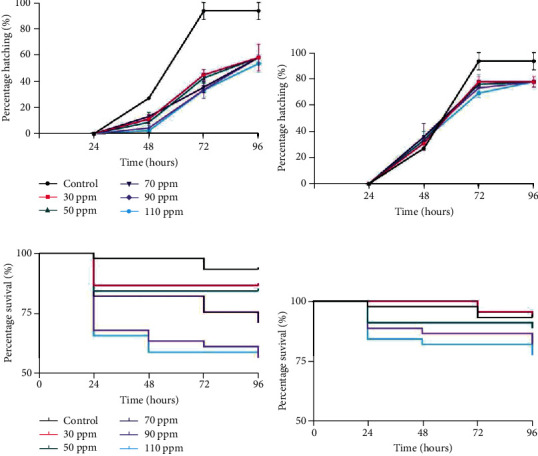
Ramification of RB4 dye and bioremediated dye on embryo hatching and survival rates. (a, b) Percentage hatching of embryos reared at different concentrations of untreated dye and treated dye, respectively. (c, d) Kaplan-Meier survival curves for zebrafish embryos reared at different concentrations of untreated dye and treated dye, respectively.

The zebrafish (*Danio rerio*) has immense advantages like small size, short life cycle, ease of breeding and maintenance, genetic similarities with humans, and high fecundity as a model system. It has been widely used as an effective biomarker in environmental toxicology. Zebrafish embryo serves as an alternative to the higher vertebrate model for which ethical consideration has become more contentious [32], thus gaining immense popularity in revealing the repercussion of natural or man-made chemicals [17]. In the FET test, control embryos showed a normal growth pattern, while a constant abnormal hatching pattern was observed in the case of test samples from 30 ppm to 110 ppm. Embryos reared in treated dye showed equivalent hatching rate as control whereas the ones in untreated dye exhibited a slightly delayed hatching at all concentrations under consideration (Figures 5(a) and 5(b)). At 96 hpf, embryos in untreated dye showed around 58% hatching (30, 50, 70, and 90 ppm) and 53% hatching for 110 ppm dye, whereas embryos at all the concentrations of treated dye showed 78% hatching, which accounts for nearly 26% amelioration in toxicity posttreatment of dye using HRs. This delayed hatching of the eggs can be attributed to abridged expression of hatching-specific enzymes and embryonic movements that reduce the ability of the embryo to break the egg envelope as reported for other RB dyes [7]. Heart rate was also measured after 24 hpf till 96 hpf, but no significant difference was observed between control and both types (untreated and treated) of test samples (data not shown).

Zebrafish mortality is another important parameter to account for the dye toxicity. The untreated dye sample was observed to affect embryo survival percentage, even at the lowest concentration, but a sharp decline was observed at 70 and 90 ppm, i.e., 71.1% and 5%, respectively. Least survival percentage was noted in 110 ppm of untreated dye which seems to be quite close to the survival percentage of 90 ppm dye-exposed embryos. This major drop of the survival curve in 70 and 90 ppm of untreated dye seems to get flattened at 110 ppm. Upon bioremediation, improvement in the embryo survival was observed as shown in Figures 5(c) and 5(d). The survival percentage in 70 and 90 ppm of treated dye was recorded to be 88.9 and 82.2%, whereas the highest concentration of 110 ppm treated dye showed 77.8% survival. Therefore, the toxicity induced by untreated dye seems to be significantly mitigated indicating the promising nature of HRs in the bioremediation of textile dyes.

**Figure 6:**
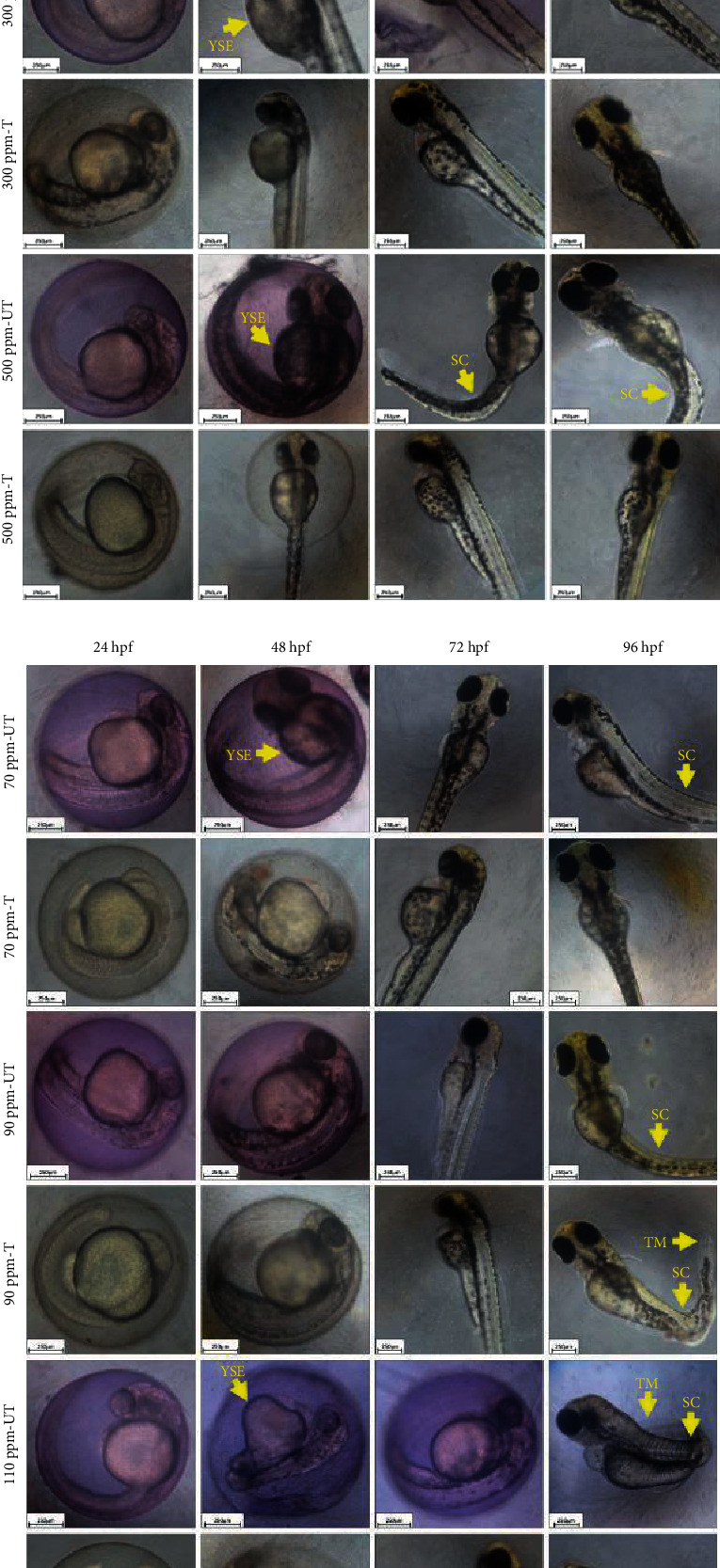
(a) Photomicrograph representing morphological abnormalities in zebrafish caused by RB4 dye and bioremediated dye exposure at various concentrations (0-50 ppm). The dashed yellow line represents the normal spinal axis. YSE: yolk sac edema; SC: spinal curvature. (b) Photomicrograph representing morphological abnormalities in zebrafish caused by RB4 dye and bioremediated dye exposure at various concentrations (70-110 ppm). The dashed yellow line represents the normal spinal axis. YSE: yolk sac edema; SC: spinal curvature; TM: tail malformation.

Zebrafish embryos were monitored at every 24 h intervals till 96 hpf. A striking difference between the growth patterns was observed between the embryos reared at all the concentrations of untreated dye and treated dye (Figures 6(a) and 6(b)). Embryos raised at 50 ppm dye and above showed delayed hatching and other morphological deformities both before and after the bioremediation of RB4. 90% of the embryos displayed hyperpigmentation at 72 hpf and 96 hpf, respectively, for untreated dye samples (90 ppm and 110 ppm), while the extent of hyperpigmentation was reduced to around 50% of embryos after dye treatment. Though yolk sac edema (YSE) was prevalent in the embryos in untreated dye samples (except for 70 ppm), pericardial edema was absent. Bioremediation of RB4 ameliorated YSE, as YSE was absent in the embryos in treated dye samples. No deformity germane to somite formation was observed. A prominent spinal curvature (SC) was visible in embryos at 72 hpf and 96 hpf of 50 ppm and 70 ppm (96 hpf) untreated samples. This SC was reversed upon dye bioremediation. But SC observed at 96 hpf of 90 ppm and 110 ppm was irreversible. Lastly, tail malformation (TM) was observed in 50% embryos reared at 70 ppm at 96 hpf and in 90% embryos reared at 90 ppm and 110 ppm of untreated dye samples. At 110 ppm and 96 hpf, the embryo displayed entangling of the tail around the embryo axis. This may happen due to the defects at the molecular level which involve malformation of the tail and motor proteins required for the tail movement. Bioremediated dye samples displayed better results while nullifying TM for 70 ppm at the same time interval and reducing the deformity to 10% in the case of 90 ppm and 110 ppm samples. To the best of our knowledge, the toxicity of RB4 on the molecular cell signaling of zebrafish is not available. It is previously known that zebrafish T-box genes namely *spadetail* (*spt*) and *no tail* (*ntl*) are involved in the formation of the medial floor plate that in turn gives rise to the tail where *pipetail* (*ppt*) and *kugelig* (*kgg*) play an important role [33, 34]. Other textile azo dyes, namely, DB38, RO16, and DR28, induce toxicity in the early developmental stages of zebrafish that includes the curved tail, delayed hatching, and YSE, respectively [16]. Therefore, in the same context, RB4 could be attributed to interfering with the tail formation pathway in zebrafish embryos by influencing the expression of these proteins.

### 3.5. Biochemical Assays

**Figure 7:**
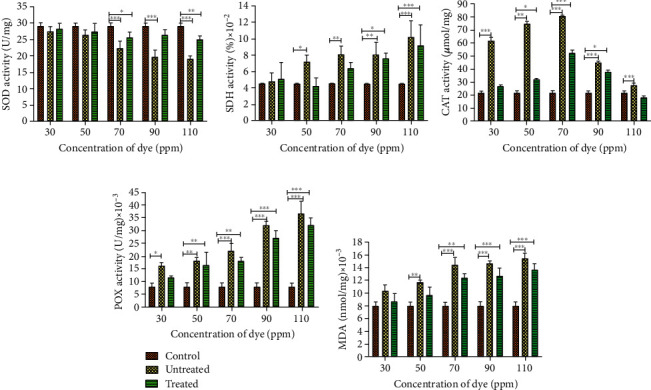
Oxidative stress profile of zebrafish larva exposed to untreated and treated dye: (a) SOD; (b) SDH; (c) CAT; (d) POX; (e) LPO.

If the rate of reactive oxygen species (ROS) formation is greater than the rate of their elimination, then the inactivation of enzymes, damage of the DNA, and peroxidation of unsaturated fats can further destroy the integrity of the cell [35]. The activities of enzymes like SOD, CAT, and glutathione peroxidase (GPx) are used as redox biomarkers under oxidative stress to measure the status of scavenging capacities that can nullify ROS generated by environmental contaminants [36]. In this study, activities of SOD, SDH, CAT, POX, and MDA levels in zebrafish larvae were investigated in treated and untreated dye samples (Figure 7). The SOD activity in untreated samples showed a declining trend with a significant decrease of ~1.2-fold at 70 ppm (*p* < 0.001) which continued to decline by ~1.5-fold at 110 ppm, which may be attributed to a high level of oxidative stress generated beyond the preventive potential of the existing SOD level [37]. Similar decreasing trends in SOD activities are reported wherein zebrafish larvae were exposed to increasing dimethyl phthalate concentration [38] or pesticide endosulfan [39]. In treated samples, SOD activity was found to be relatively closer to the control for initial concentrations and marginal decline at the maximum concentration, i.e., 110 ppm (*p* < 0.01). The higher levels of SOD in treated samples (i.e., 70-110 ppm), compared to untreated samples, suggest that to protect cells from free radicals, more proteins are required that bolster the enzymatic activity against oxidants [40]. SDH activity was found to increase in both untreated and treated dyes with respect to the control (Figure 7(b)). However, samples treated with 110 ppm of untreated and treated dye showed an increased SDH activity by 2.38-fold and 2.26-fold, respectively, as compared to control. In untreated samples, the relative concentration of H_2_O_2_ was slightly higher as compared to the treated samples (*p* < 0.01). Due to low levels of SDH in the untreated dye, there was an escalated production of H_2_O_2_. Excess H_2_O_2_ buildup due to SOD and SDH activity becomes toxic to cells. As CAT and POX enzymes catalyze the conversion of H_2_O_2_ to molecular oxygen and water, the activity of these enzymes was evaluated. A change in CAT activity after exposure to dye is shown (Figure 7(c)). At lower concentrations of untreated dye (30-70 ppm), there was a significant increase (*p* < 0.001) in CAT activity by 3-, 3.75-, and 4-fold, respectively, in comparison to control, in order to reduce or counterbalance the excessive ROS production. But at 90 and 110 ppm of dye exposure, there was a significant reduction in CAT activity (*p* < 0.05). After a threshold concentration of dye exposure, the CAT could no longer eliminate the increased oxidative stress generated, and thus, CAT activity reduces [38]. In another study, Meireles et al. reported a decline in the CAT activity to 1.55-, 1.44-, and 1.25-fold when Red Disperse dyes, i.e., DR60, DR73, and DR78, were used, respectively [41]. According to Cong et al., exposure to a higher concentration of dye can cause oxidative damage leading to reduced CAT activity, consequentially depleting CAT and SOD enzymes [38]. In our study, at the lowest concentration of treated dye exposure (30 ppm), there was a negligible increase in CAT, and at a higher concentration, i.e., 70 ppm, activity was found to have increased significantly (*p* < 0.001) by ~2.5-fold as compared to control. With a further increase in dye concentration (>70 ppm) of treated samples, a significant decline (*p* < 0.001) was observed. This investigation showed remarkably low levels of CAT activity in treated samples when compared to untreated samples from 30 to 110 ppm. This can occur in view of SOD enzyme dysfunction caused by stress-induced inactivation of its active site [42] in untreated dye. In Figure 7(a), SOD levels in untreated samples were reduced as compared to treated samples, leading to curtailed H_2_O_2_ production, and may thus result in deficiency of CAT synthesis in untreated dye. POX catalyses the removal of H_2_O_2_ by oxidising a substrate pyrogallol to purpurogallin [43]. POX activity in both treated and untreated samples produced a rising trend as compared to control (Figure 7(d)). For any given concentration, the POX activity of untreated dye was distinctly higher than that of treated dye. A higher POX activity is a consequence of high H_2_O_2_ levels. Increased POX is associated with tissue damage and can lead to disruption in larval development [44]. The lower POX activity in treated samples may occur due to minimized oxidative stress in treated samples. It is therefore noted that these antioxidant enzymes are adept at capturing H_2_O_2_ and superoxide anions which lead to the protection of organisms from oxidative stress conditions [45].

Free radicals induce lipid peroxidation, wherein degradation of lipid peroxides leads to the production of many subproducts including MDA, the levels of which can be used to determine the severity of oxidative damage evoked in larvae [46]. The untreated dye exhibits a rising trend in MDA levels with a significant increase of ~1.7-fold (*p* < 0.001) at 70 ppm with respect to control (Figure 7(e)). At lower concentrations (i.e., 30-50 ppm), the level of MDA was relatively closer to control, but at a slightly higher concentration, i.e., 70 ppm, ~1.5-fold rise in MDA levels was observed. In the presence of high dye toxicity (i.e., 90 ppm onwards), the ability of antioxidant enzymes to eliminate ROS reduces, and therefore, the residual free radicals attack unsaturated fatty acids inciting an increase in MDA content of larvae [46]. Analogous results were observed using naphthalene sulfonic acid (NSA), metanilic acid (MA), and acid blue 113 (AB113) textile dye [47] and fungicide azoxystrobin [48], where an apparent rise in MDA content of zebrafish was paralleled with an increase in concentration. A similar observation was reported by Mao et al. using pesticides [49]. In our study as well, the content of MDA in treated samples is fairly reduced as compared to that in untreated RB4 samples. This suggests the occurrence of minimized MDA formation or the ability to scavenge them as an important measure for preventing cell damage in hairy root bioremediated samples. Similar results were proclaimed in a study by Cong et al., where oxidative damage in fish, treated with a low concentration of DMP for 24 h, was effectively exterminated by the cause of its antioxidant mechanisms [38].

In the untreated samples, the concentration of dye, i.e., 50 ppm onwards, causes severe damage in embryos. But in the treated samples of 50 and 70 ppm, the activity of antioxidant enzymes such as SOD, SDH, CAT, POX, and LPO content (Figure 7) indicates toxicity amelioration after treatment with HRs as there was delayed hatching, morphological deformities, and the absence of YSE in zebrafish embryos. These enzymes have been known to play an important role in early stress even at relatively low concentrations of dye, helping to reduce ROS [50]. However, at a further increase in the concentration of dye, i.e., 90 and 110 ppm, the ROS produced is also high, which cannot be remediated by HRs, in the severity of stress like embryo hyperpigmentation. These may be due to stress-induced inhibition or changes in the subunit of the antioxidant enzymes [51]. Several reports have shown the toxic nature of different anthraquinone dyes, but to the best of our knowledge, this is the first report on comprehending the repercussion of untreated and bioremediated RB4 dye on zebrafish embryos thereby contributing to the rudimentary understanding of the impact of remediated RB4 on ecosystems.

## 4. Conclusion

In summary, we emphasize the need to develop nontoxic dyes or efficient methods to treat industrial effluents having synthetic dyes. The HRs used in this study were found to be effective in the degradation of potentially toxic textile dyes, and treated dye was found to be less toxic in comparison to untreated, after in vivo toxicity assessment. We also hypothesize that RB4 might be hindering the normal cellular pathway for tail formation further contributing to the increased mortality in embryos. The expression of different antioxidant enzymes during dye exposure corroborates the synchronous activity of antioxidant machinery to protect developmental toxicity in zebrafish embryos. This study thus suggests that HRs can be accounted as viable candidates for the treatment of RB4-contaminated effluents. This study has added novel approaches of remediation-based investigation to understand the activity of enzymes under dye stress in a process to develop a system for degradation of high dye concentration with minimal or no residual toxicity.

## Figures and Tables

**Table 1:** Phytodegradation analysis of RB4 and its degradation products.

	Number of cells examined	Average number of roots	Mitotic index (MI)	% aberrant
Distilled water	510	14 ± 1.16	9.8 ± 0.52	4.0 ± 1.15
Untreated (110 ppm)	470	8 ± 1.15^ns^	6.5 ± 1.10^ns^	38 ± 0.55^a^^∗∗∗^
Treated (110 ppm)	480	12 ± 1.15^ns^	8.5 ± 0.55^ns^	10 ± 1.15^aNS,b^^∗∗^

The data represent mean ± SD. Symbols in the figure represent that comparisons are made between ^a^sample dye vs. control (*p* < 0.001) and ^b^treated dye vs. untreated (*p* < 0.01). Statistical significance: ^∗∗∗^*p* < 0.001 and ^∗∗^*p* < 0.01. NS: nonsignificant.

## Data Availability

The data used to support the findings of this study are included within the article.

## Supplementary Materials

Supplementary Materials Figure S1: (A) untreated dye, (B) treated dye (with HRs) at 0 h, and (C) treated dye (with HRs) at 120 h. Figure S2: effect of different pH on % decolourization by HRs. Figure S3: effect of different temperatures on % decolourization by HRs. Figure S4: effect of different biomass dosages on % decolourization by HRs. Figure S5: effect of different concentrations of dye on % decolourization by HRs.
