# Supplementary material for: Toxicity Mitigation of Textile Dye Reactive Blue 4 by Hairy Roots of Helianthus annuus and Testing Its Effect in In Vivo Model Systems
Source: Biomed Res Int. 2022 Jul 25;2022:1958939. doi: 10.1155/2022/1958939 (PMC9343192; doi:10.1155/2022/1958939)
Supplement: Supplementary Materials — Figure S1: (A) untreated dye, (B) treated dye (with HRs) at 0 h, and (C) treated dye (with HRs) at 120 h. Figure S2: effect of different pH on % decolourization by HRs. Figure S3: effect of different temperatures on % decolourization by HRs. Figure S4: effect of different biomass dosages on % decolourization by HRs. Figure S5: effect of different concentrations of dye on % decolourization by HRs. [file 1958939.f1.docx]

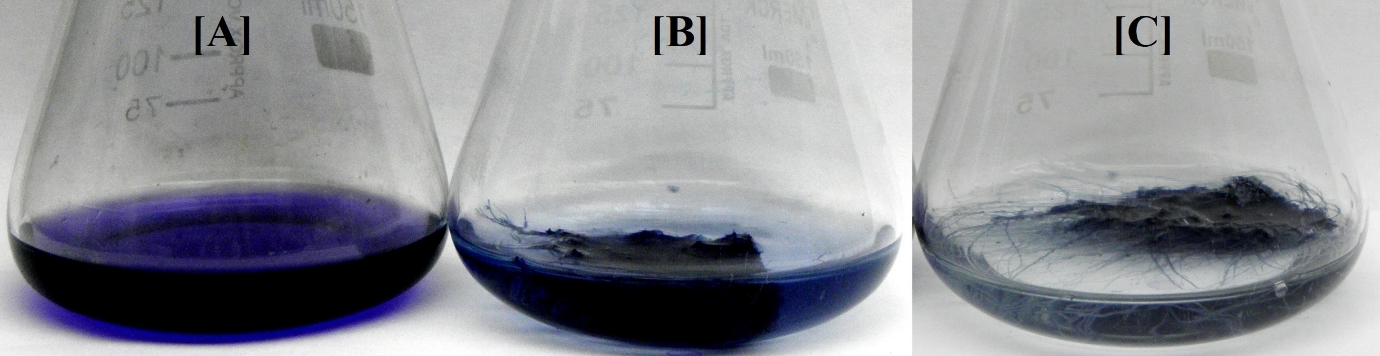


Fig. S1. A – Untreated dye, B- Treated dye (with HRs) at 0h , C- Treated dye (with HRs) at 120h


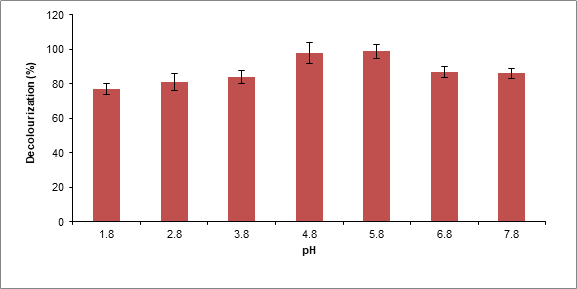


Fig. S2. Effect of different pH on % decolourization by HRs


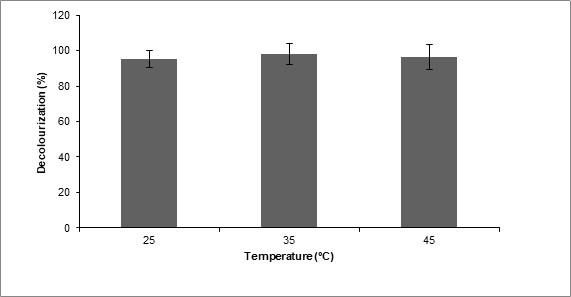


Fig. S3. Effect of different temperature on % decolourization by HRs


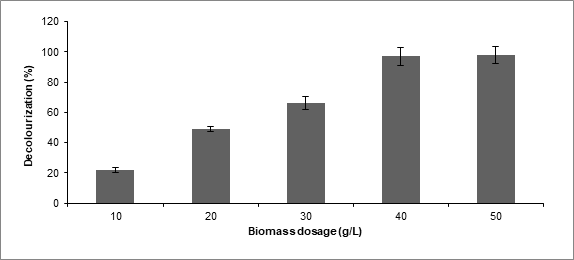


Fig. S4. Effect of different biomass dosage on % decolourization by HRs


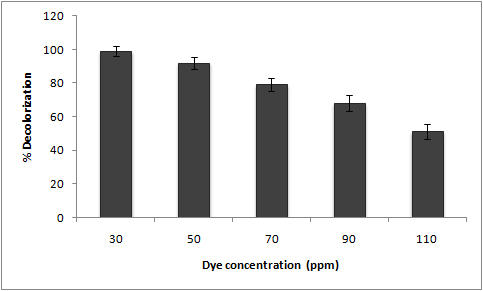


Fig. S5. Effect of different concentrations of dye on % decolourization by HRs
